# N-Linked Glycosylation Supports Cross-Talk between Receptor Tyrosine Kinases and Androgen Receptor

**DOI:** 10.1371/journal.pone.0065016

**Published:** 2013-05-28

**Authors:** Harri M. Itkonen, Ian G. Mills

**Affiliations:** 1 Prostate Cancer Research Group, Centre for Molecular Medicine Norway, Nordic European Molecular Biology Laboratory (EMBL) Partnership, University of Oslo and Oslo University Hospital, Oslo, Norway; 2 Department of Cancer Prevention and Department of Urology, Oslo University Hospitals, Oslo, Norway; Florida International University, United States of America

## Abstract

Prostate cancer is the second most common cause of cancer-associated deaths in men and signalling via a transcription factor called androgen receptor (AR) is an important driver of the disease. Androgen treatment is known to affect the expression and activity of other oncogenes including receptor tyrosine kinases (RTKs). In this study we report that AR-positive prostate cancer cell-lines express 50% higher levels of enzymes in the hexosamine biosynthesis pathway (HBP) than AR-negative prostate cell-lines. HBP produces hexosamines that are used by endoplasmic reticulum and golgi enzymes to glycosylate proteins targeted to plasma-membrane and secretion. Inhibition of O-linked glycosylation by ST045849 or N-linked glycosylation with tunicamycin decreased cell viability by 20%. In addition, tunicamycin inhibited the androgen-induced expression of AR target genes KLK3 and CaMKK2 by 50%. RTKs have been shown to enhance AR activity and we used an antibody array to identify changes in the phosphorylation status of RTKs in response to androgen stimulation. Hormone treatment increased the activity of Insulin like Growth Factor 1-Receptor (IGF-1R) ten-fold and this was associated with a concomitant increase in the N-linked glycosylation of the receptor, analyzed by lectin enrichment experiments. Glycosylation is known to be important for the processing and stability of RTKs. Inhibition of N-linked glycosylation resulted in accumulation of IGF-1R pro-receptor with altered mobility as shown by immunoprecipitation. Confocal imaging revealed that androgen induced plasma-membrane localization of IGF-1R was blocked by tunicamycin. In conclusion we have established that the glycosylation of IGF-1R is necessary for the full activation of the receptor in response to androgen treatment and that perturbing this process can break the feedback loop between AR and IGF-1R activation in prostate cells. Achieving similar results selectively in a clinical setting will be an important challenge in the future.

## Introduction

Prostate cancer is the second most common cause of cancer associated deaths in men. Androgen receptor (AR) has been identified as a key driver of localised and metastatic prostate cancer and a principal therapeutic target [1], [2]. The challenge in the treatment is the development of a castration resistant disease, which still expresses AR and retains AR activity [3], [4]. AR belongs to the nuclear receptor super family and it is activated by steroid hormones, predominantly testosterone and di-hydrotestosterone [5], [6]. Ligand binding triggers nuclear translocation of the AR and consequent AR-driven gene expression. AR target genes have been established as candidate oncogenes and biomarkers in prostate cancer and in recent years chromatin immunoprecipitation coupled to high-throughputsequencing (ChIP-seq) and expression profiling has enabled an unbiased identification of AR-driven genenetworks. Pathway analysis of these networks has implicated the AR in the regulation of metabolism [7]–[9] and endoplasmic reticulum (ER) stress response [10] in prostate cancer cells.

Changes in the expression of certain AR target genes can help to sustain AR transcriptional activity [11], [12]. As an example, Insulin like Growth Factor 1-Receptor (IGF-1R) forms a regulatory feed-back loop with AR. AR itself can activate IGF-1R expression [13] and IGF-1R stimulates AR activity in prostate cancer cells [14]–[16]. Receptor tyrosine kinases (RTK) form an especially interesting group of proteins as their aberrant activation is frequently documented in other cancers, which has enabled development of targeted therapies [17]–[19]. RTKs act as receptor kinases to activate complex down-stream signalling networks. The activity of RTKs can be regulated at the transcriptional and translational levels [17], [20]. However, plasma-membrane retention time determines how long a given receptor activates signalling and is therefore a critical determinant of RTK activity [17], [21]–[23]. Plasma-membrane retention is regulated by negative feedback via mTOR [23] but also by the amount of N-linked glycosylation, occurring in the late ER and Golgi [24], [25]. The enzymes catalyzing N-linked glycosylation are sensitive to the levels of hexosamines. Hexosamine biosynthetic pathway (HBP) in turn requires glucose and glutamine, which makes this pathway capable of sensing the availability of energy. Increase in the availability of metabolites can result in an increase in HBP flux, which enables cells to drive growth-promoting programs.

Given that the AR activates metabolic networks and regulates ER functions, we hypothesized that prostate cancer cells might exhibit increased expression of HBP enzymes. This would enable cancer cells to support aberrant growth promoting signalling and AR activity. We used an antibody array to identify changes in the phosphorylation status of RTKs during androgen stimulation. We then found that androgen-induced changes in glycosylation of RTKs are important for processing of these receptors. Inhibition of glycosylation blocks hormone-dependent activation and affects subcellular distribution of the receptors. Significantly we have found that inhibiting glycosylation even in conditions where the AR is active can effectively inhibit the downstream AR-dependent translation of target genes. This represents the first study in prostate cancer cells to attempt to disrupt the feedback loop between hormones and RTK activation by targeting glycosylation and confirms the importance of this process.

## Materials and Methods

All chemicals and reagents were obtained from Sigma Aldrich unless otherwise stated.

### Cells lines and maintenance

Cells were maintained according to provider's guidelines. LNCaP (CRL-1740), VCaP (CRL-2876), PC-3 (CRL-1435) and RWPE-1 (CRL-11609) cell-lines were obtained from the American Tissue Culture Collection and the PNT2-cell line (95012613) was obtained from Sigma Aldrich. Prior to R1881 stimulation, cells were maintained in phenol-red free media supplemented with 10% charcoal stripped serum for 72 hours.

### Treatments and viability assays

Synthetic androgen, R1881 (Sigma Aldrich), was solubilised in ethanol to a final concentration of 10 µM and unless otherwise stated, used in a 10 nM dose in cell lines, as previously reported [7], [26]. Inhibitor against human O-GlcNAc transferase, ST045849 was purchased from TimTec (Newark, USA) and solubilised in DMSO to a final concentration of 20 mM. Tunicamycin was obtained from VWR (654380-10) and solubilised in DMSO.

Viability was assessed using CellTiter-Glo reagent (G3581, Promega, Stockholm, Sweden) according to manufacturer's instructions. Experiments were repeated three times, with six technical replicates on each occasion. The statistical analysis was performed based on the average values of these three independent experiments by Student's T-test.

### Preparation of cell lysates and western blot

All the steps were performed at 4°C. Cells were washed once with PBS and harvested in cell lysis buffer (10 mM Tris-HCl pH 8.0, 1 mM EDTA, 0.5 mM EGTA, 1% TritonX-100, 0.1% Na-deoxycholate, 0.1% SDS, 140 mM NaCl + Complete protease inhibitor mixture (11836170001, Roche), rotated for 15 minutes, sonicated ((20 cycles, 30 seconds on/30 seconds off) using a Bioruptor (Diagenode, Belgium) and centrifuged at 18000x*g* for 5 minutes. Supernatant was collected and protein concentration determined with BCA Protein assay kit (23227, VWR). 10–25 µg of lysate was separated with SDS polyacrylamide gel electrophoresis, using 4–12% gradient gels (NP0323, Invitrogen) and transferred to nitrocellulose membranes (IB301002, Invitrogen). Membranes were probed with antibodies against GFPT1 (#3818), IGF-1R (#3027), EGFR (2232S), Cox4 (#5247S), BiP (c50b12), GAPDH (#3683S) and Actin (#5125S) (Cell Signalling Technology), ErbB2 (ab16901), UAP1 (HPA014659, Sigma), KLK3 (A0562, Dako). Primary antibodies were detected with HRP-conjugated secondary antibodies against cognate species (anti-rabbit P0448 and mouse P0447; Dako). The intensities of the signals arising from Western blot were quantified with the Quantity One software (Bio-Rad).

### Immunoprecipitation and lectin pulldown

All the steps were performed at 4°C. Cells were washed once with PBS and solubilized in cell lysis buffer (10 mM Tris-HCl pH 8.0, 1 mM EDTA, 0.5 mM EGTA, 1% TritonX-100, 0.1% Na-deoxycholate, 0.1% SDS, 140 mM NaCl + Complete protease inhibitor mixture, Roche), rotated for 15 minutes and centrifuged 18000 g for 5 minutes. Protein concentration was determined with BCA assay and 1000–3000 µG of protein was pre-cleared with unspecific antibody (sc-2027, Santa Cruz) and protein A-coated magnetic Dynabeads (Invitrogen) for immunoprecipitation (IP) or un-bound agarose beads (AG1000, VectorLabs) for lectin pulldown for 2 hours. Pre-cleared extract was used was for IP and lectin pulldown (Phaseolus vulgaris Leucoagglutinin, L-PHA, AL-1113, VectorLabs) over night. Protein A-coated magnetic beads were added to the IP reaction, incubated for two hours and washed with IP wash buffer (0.5% NP-40, 150 mM NaCL, 20 mM Tris-HCl, pH 8.0). Lectin pulldown was washed three times with lectin wash buffer (0.1% Tween, 150 mM NaCl, 10 mM Tris-HCl, pH 8.0).

### Receptor tyrosine kinase antibody array

The array experiment was performed according to manufacturer's instructions (ARY001B, R&D systems). The intensity of the signals from each antibody was quantified by Quantity One software (Bio-Rad). Specific signals were normalized against signals arising from unspecific antibodies printed as reference controls on the array.

### RT-PCR

RNA was isolated with illustra RNAspin Mini Kit (25-0500-70, GE Healthcare) and cDNA was produced by qScript™ cDNA Synthesis Kit (95047-025, Quanta Biosciences). Quantitative PCR (qPCR) was performed using Power SYBR Green PCR master mix (4385612, Applied Biosystems) or TaqMan Universal PCR master mix (4369016, Applied Biosystems) on a 7900HT Fast Real-Time PCR system (Applied Biosystems). Primers for SYBR green assays for EGFR, Her2 and IGF-1R were (R-CGCAAGTGTAAGAAGTGCGAA, F-GTAGCATTTATGGAGAGTGAGTCT), (R-AGGGAGTATGTGAATGCC, F-GGCCACTGGAATTTTCAC) and (F- GCGTGAGAGGATTG, R-CTTATTGGCGTTGAGGTATGC) and TBP, CaMKK2 and KLK3 were measured using TaqMan assays (4326322E, hs00198032, Hs02576345_m1, respectively, Applied Biosystems).

### Identification of putative AR and Pol2 binding sites

We took an *in silico* approach to find if AR and RNA polymerase II (RNA polII) associate with specific genomic loci of interest. We utilized two publicly available ChIP-seq datasets and visualized the data using the UCSC Genome Browser (AR, accession number GSE14092 [7] and RNA polII, accession number GSE28126 [8]).

### Immunofluorescence

LNCaP cells were plated on cover slips and allowed to attach for 48 hours. At this point cells were treated as described above for R1881 stimulation. Cells were fixed with ice-cold methanol and placed in −20°C overnight. On the next day, cells were washed twice with PBS and once with 5% BSA in PBS. Subsequently, the cells were blocked with 5% BSA in PBS for an hour, followed by an incubation with a primary antibody (1∶50) against IGF-1R for an hour. Coverslips were washed three times with 5% BSA in PBS and stained with a secondary antibody (A-11010, Invitrogen) and DAPI staining for an hour. After this, cover slips were washed three times with PBS and mounted with fluorescent mounting media (S3023, DAKO). Imaging was performed with Zeiss LSM 510 confocal microscope.

## Results

### Hexosamine biosynthetic pathway is up-regulated in prostate cancer cell lines

**Figure 1:**
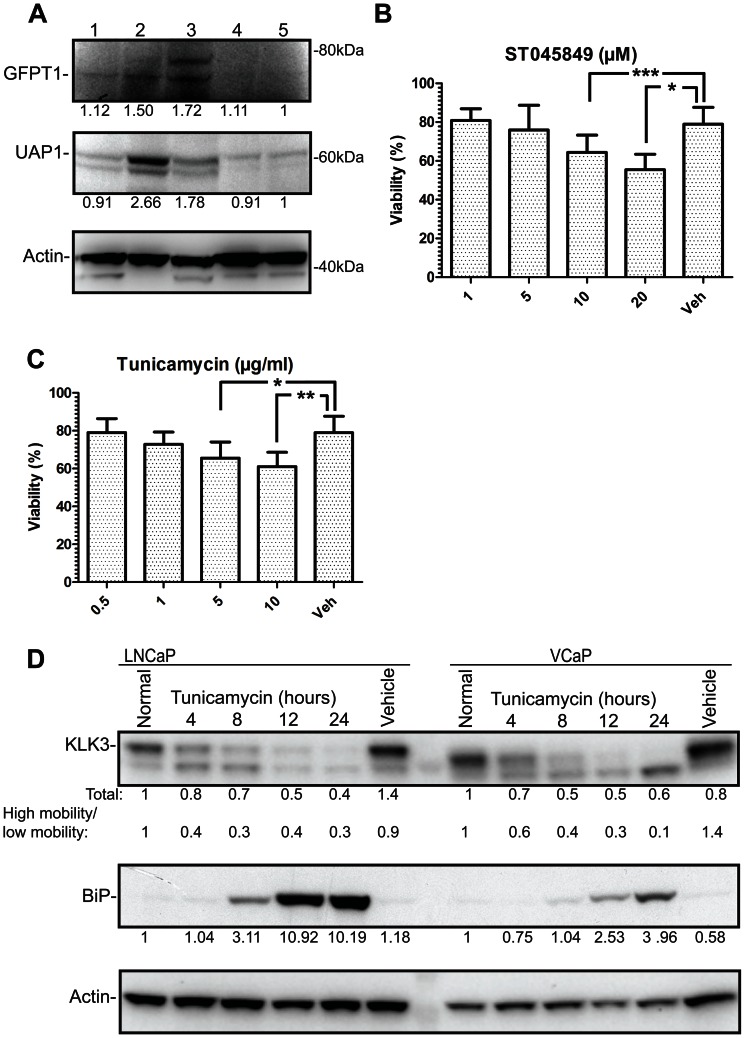
Hexosamine biosynthetic pathway is up-regulated in prostate cancer cell lines. A) Cytosolic fractions were collected and analyzed by Western blot. Expression levels of the hexosamine biosynthetic pathway (HBP) proteins in cell lines representing prostate cancer (lane 1:PC3, lane 2:LNCaP and lane 3:VCaP) and benign prostate epithelial cells (lane 4:PNT2 and lane 5:RWPE-1). The intensity of the WB signal was determined by densitometry, normalized to actin and the amount in RWPE-1 cells was set to one. This experiment was repeated twice. LNCaP cells were treated either with a concentration gradient of OGT inhibitor, ST045849 (B) or an inhibitor of N-linked glycosylation, tunicamycin (C). Viability was assessed after 48 hours using an MTS assay and values were normalized to sample without any treatment. Assays were repeated three times, each time six technical replicates and SEM of the biological replicates is shown. Statistical analysis was performed with Student's t-test, (*<0.05, **<0.01, ***<0,001). D) LNCaP and VCaP cells were treated with tunicamycin (5 µg/ml) for the indicated time and protein lysates were harvested. The intensities of KLK3 and BiP signals were determined with densitometry, normalized to actin and the protein amount in normal condition was set to one. The relationship between the high and low mobility KLK3 is shown. This experiment was repeated twice.

The rate-limiting enzyme in the hexosamine biosynthetic pathway (HBP) is glutamine-fructose-6-phosphate transaminase 1 (GFPT1), which is also the first enzyme in the pathway [27]. UDP-N- acetylglucosamine pyrophosphorylase 1 (UAP1) is the final enzyme in the pathway producing UDP-N-Acetylglucosamine (UDP-GlcNAc). Interestingly, the expression of both GFPT1 and UAP1 was increased by ∼50% in AR-positive prostate cancer cell lines LNCaP and VCaP compared to normal prostate cells PNT2 and RWPE-1 (Fig. 1A). UDP-GlcNAc is utilized by O-GlcNAc transferase [28], to modify a multitude of target proteins by O-linked glycosylation [27]. In addition, UDP-GlcNAc is utilized by the ER and Golgi resident enzymes to modify proteins targeted to the plasma membrane [25].

In order to test the importance of glycosylation for prostate cancer cells, we utilized small molecule inhibitors against O-linked glycosylation (ST045849) and N-linked glycosylation (tunicamycin). ST045849 inhibits OGT [29], while tunicamycin inhibits the enzymes catalyzing N-linked glycosylation, and it is also known to induce ER stress [30]. ST045849 caused a concentration-dependent decrease in the viability of LNCaP cells and the highest concentration (20 µM) decreased viability by 20% compared to the vehicle treated cells used as control (Fig. 1B). Tunicamycin also caused reduction in viability of maximally 20% versus the vehicle treated cells (Fig. 1C). O-linked glycosylation was recently shown to be elevated in prostate cancer and OGT itself was shown to regulate invasion and angiogenesis [31]. However, the role of N-linked glycosylation in prostate cancer has been less investigated.

### Inhibition of N-linked glycosylation impairs the expression of androgen receptor target genes

Tunicamycin dose-dependently inhibited the viability of LNCaP prostate cancer cells. AR activity has been shown to induce ER stress response [10] and N-linked glycosylation occurs in the endoplasmic reticulum and Golgi. In order to determine whether N-linked glycosylation alters AR activity, we evaluated the effect of tunicamycin on the expression of Kallikrein 3 (KLK3), which is a direct AR target protein, and also a well-characterized biomarker for prostate cancer and known glycoprotein [32]. Tunicamycin treatment reduced KLK3 protein by 50% in LNCaP and VCaP cells at 12 hours after addition of tunicamycin (Fig. 1D). The predominant effect of tunicamycin treatment was to alter the ratio of high and low-molecular weight KLK3 over time, which was seen more drastically in VCaP cells. Tunicamycin induced accumulation of an ER stress marker, 78 kDa glucose-regulated protein (BiP) [33] from eight hours onwards, following the accumulation of un-processed KLK3 which was detected from four hours onwards (Fig. 1D).

We noted that inhibition of N-linked glycosylation led to the significant accumulation of a shorter, un-glycosylated form of KLK3 [34], [35] in VCaP cells (90%), while in LNCaP cells the initial accumulation of the un-glycosylated form was followed by 60% decrease of the total KLK3 (Fig. 1D). This suggests that tunicamycin mainly exerts its effects on processing in VCaP cells, while in LNCaP cells it has effects not only on processing but also on transcription. In order to test this, we stimulated LNCaP cells with a synthetic androgen in the presence or absence of tunicamycin and found that tunicamycin decreased the androgen-dependent expression of both KLK3 and another AR target gene, CAMKK2 [8], mRNAs by over 50% (Fig. S1).

**Figure 2:**
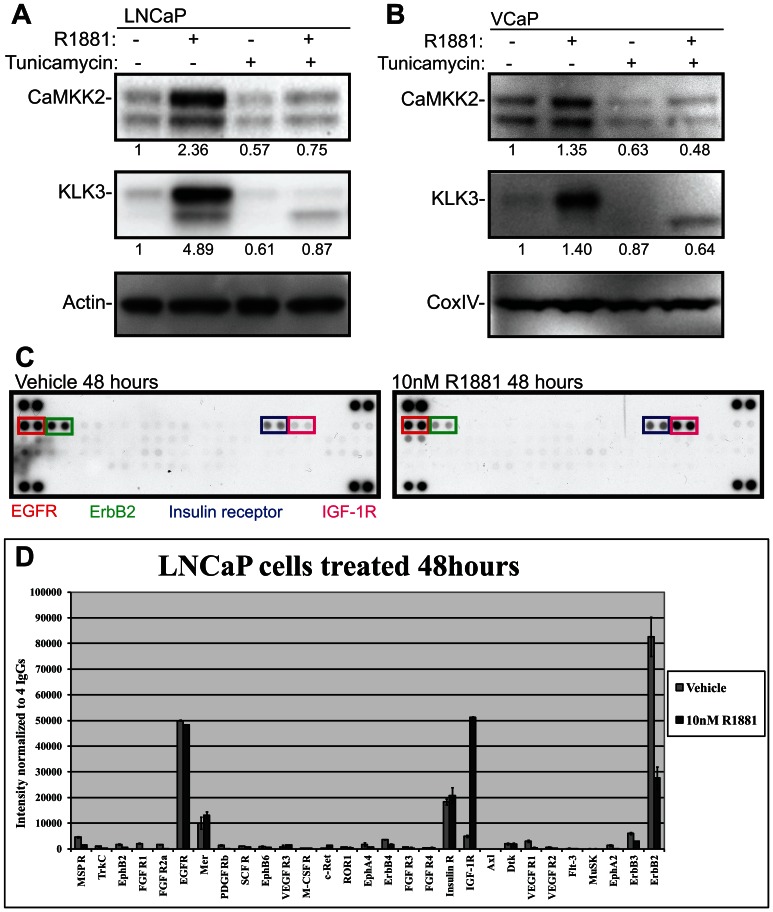
Receptor tyrosine kinases are regulated by the androgen receptor. Cells were deprived of androgens for 72 hours prior to stimulation with a synthetic androgen (10 nM R1881) for24 hours. LNCaP (A) and VCaP (B) cells were stimulated with R1881 in the presence and absence of tunicamycin (5 µg/ml) for 24 hours and protein lysates were harvested. The intensities of KLK3 and CaMKK2 were determined with densitometry, normalized to actin (LNCaP) or CoxIV (VCAP) and the protein amount in the vehicle treated cells was set to one. This experiment was repeated twice. C) LNCaP cells were stimulated with R1881 for 48 hours. Cell lysates were harvested and analyzed with a human phospho-RTK array. D) The signals arising from the array were determined with densitometry and normalized to the signals arising from the negative controls printed on the array. The RTK array data was obtained from a single experiment. Phosphorylated receptors are colour-coded in the figure.

In order to test more directly whether N-linked glycosylation affects AR activity, we stimulated LNCaP and VCaP cells with a synthetic androgen in the presence and absence of tunicamycin for 24 hours. We observed significant a 50% decrease in the expression of KLK3 and CaMKK2, upon androgen treatment in both cell-lines (Fig. 2A and 2B). This in spite of the fact that CaMKK2, unlike KLK3 is not a known target for N-linked glycosylation and the protein resides in the cytoplasm. This data suggested that tunicamycin can act not only by affecting glycosylation/post-translational processing but also through a feedback loop that affects the transcriptional activity of the AR.

### Insulin like Growth Factor 1-Receptor is activated by AR

Signalling cross-talk between kinases and the AR have been reported as one general mechanism for sustaining AR activity. In addition it has previously been reported that RTKs are extensively modified via N-linked glycosylation in the Golgi network prior to the insertion into the plasma membrane [24] and signalling cross-talk between RTKs and the AR has been reported to contribute to metastatic disease [36]. Furthermore RTKs, such as insulin growth factor 1-receptor (IGF-1R) are expressed in response to androgen treatment [13]. Together this establishes the hypothesis that the androgen-dependent expression and glycosylation of RTKs may help to maintain AR activity and the expression of AR target genes.

**Figure 3:**
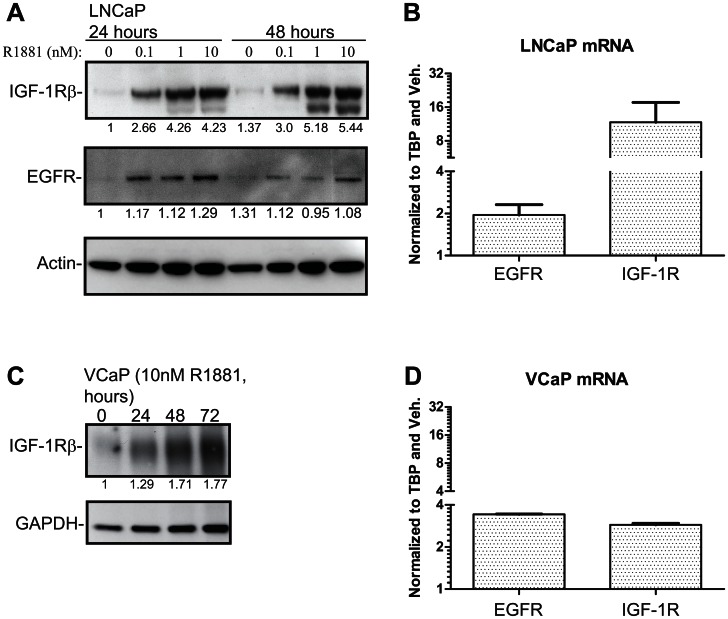
Androgen receptor regulates expression of receptor tyrosine kinases in LNCaP and VCaP cells. Cells were deprived of androgens for 72 hours prior to stimulation with a synthetic androgen (R1881) for the indicated time. A) LNCaP cells were treated with a concentration gradient of R1881, protein lysates were harvested at 24 and 48 hours and samples analysed by WB. The intensity of IGF-1R and EGFR bands were determined with densitometry, normalized to actin and the protein amount in the vehicle treated sample was set to one. Experiment was repeated twice. B) LNCaP cells were stimulated with either 10 nM R1881 or vehicle and total mRNAs were collected after 18 hours. Values were first normalized to TBP and then to the vehicle treated sample. The data shown represents values obtained from a biological replicate and standard error of mean shown. C) VCaP cells were treated with 10 nM R1881 and protein lysates were harvested at the indicated time points. The intensity of the bands was determined with densitometry, normalized to GAPDH and the Western blot signal at the zero hours timepoint was set to one. D) VCaP cells were treated with 10 nM R1881 and mRNA samples were harvested 18 hours after the stimulation. The data shown represents values obtained from a biological replicate and standard standard error of mean is shown.

To identify candidate RTKs as mediators of this effect we used an antibody array covering a panel of RTKs. LNCaP cells were stimulated with a synthetic androgen for 48 hours to allow changes in AR-dependent proteome and cell lysates were analyzed with the RTK array to assess RTK activity (Fig. 2C). Epidermal growth factor receptor (EGFR) phosphorylation was detectable and unchanged both in the presence and absence of the hormone (Fig. 2C and 2D). By contrast, androgen stimulation led to a 3-fold decrease in the activity of ErbB2 (Her2), while signalling via IGF-1R was increased by 10-fold. Based on this approach, the RTKs most likely to have a feedback effect on AR activity are IGF-1R and ErbB2, given the reciprocal increase in the activity of the former and reduction in the activity of the latter during androgen stimulation. We confirmed that androgen treatment did indeed cause over 50% increase of IGF-1R in both LNCaP and VCaP cells at both the transcript (Fig. 3B and Fig. 3D) and protein level (Fig. 3A and Fig. 3C). This corroborates previous reports that IGF-1R is upregulated in response to androgen treatment[13]. In addition EGFR has been shown to be androgen-regulated at the protein and mRNA levels and we were able to confirm this as a control in the same experiment (Fig. 3)[37]. We also reanalysed chromatin immunoprecipitation and sequencing (ChIP-seq) data for AR [7] and RNA polII [8] from these cell-lines and found AR binding sites associated with the gene body and promoter regions of EGFR and IGF-1R in both cell lines (Fig. S2 and S3) and also hormone-dependent recruitment of RNA polII in LNCaP cells (Fig. S2A and S2B), further supporting androgen regulation of these genes. Strikingly IGF1-R is therefore an RTK which is androgen dependent at three levels: in its transcript expression, in its protein expression and in its activity. This multilevel androgen dependency made this the RTK that we focussed on for the rest of the study.

**Figure 4:**
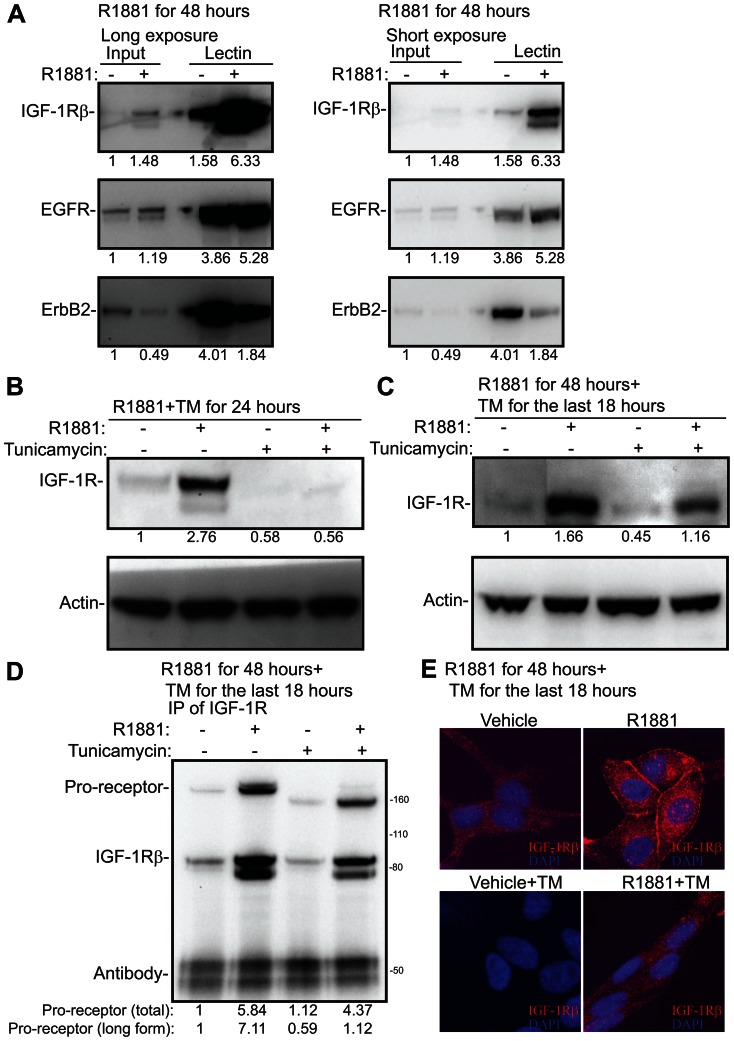
N-linked glycosylation is required for the processing and localization of Insulin like Growth Factor 1-Receptor. A) LNCaP cells were deprived of androgens for 72 hours, stimulated with 10 nM R1881 and protein lysates were harvested after 48 hours of the treatment. Phaseolus Vulgaris Leucoagglutinin lectin was used to enrich proteins modified via N-linked glycosylation from androgen stimulated cells. These enriched fractions were then analyzed by the means of Western blotting and blotted for IGF-1R, EGFR and ErbB2. Two different exposures of the same experiment are shown. The intensity of each band was determined with densitometry, normalized to the background and the input sample treated with vehicle was set to one. This experiment was repeated twice. B) LNCaP cells were deprived of androgens for 72 hours, stimulated with 10 nM R1881 for 24 hours either in the presence or absence of tunicamycin (5 µg/ml) and protein lysates were harvested. The intensity of the bands were determined with densitometry, normalized to actin and the vehicle treated sample was set to one. This experiment was repeated twice. C) LNCaP cells were deprived of androgens for 72 hours, stimulated with 10 nM R1881 for 48 hours and tunicamycin (5 µg/ml) was added for the last 18 hours where indicated. The intensity of the bands were determined with densitometry, normalized to actin and the vehicle treated sample was set to one. This experiment was repeated twice. D) LNCaP cells were treated as in C, protein lysates were harvested and IGF-1R antibody was used to immunoprecipitate (IP) the receptor. Membranes were probed with an antibody against IGF-1Rβ. The bands corresponding to IGF-1R pro-receptor, IGF-1Rβ subunit and IgG heavy chain are depicted. The density of the total pro-receptor and the longer forms were determined with densitometry and vehicle treated condition was set to one. This experiment was repeated twice. E) LNCaP cells were treated as in C, harvested for immunofluorescence by methanol fixation and stained for IGF-1R. Images were obtained with a confocal microscope with the same microscope settings for each condition. This experiment was repeated twice and representative images are shown.

The activity of RTKs can be stimulated by increased N-linked glycosylation [24], [25]. In order to test whether this is the case in prostate cancer cells, we stimulated LNCaP cells with androgen and used a lectin-based approach to enrich proteins modified via N-linked glycosylation (Fig. 4A). Interestingly, androgen stimulated activation of IGF-1R was accompanied by 3-4-fold enhanced glycosylation of the receptor. In contrast, the activity of EGFR was un-altered by the androgen treatment (Fig. 2D) and we did not observe significant changes in the androgen induced glycosylation either (Fig. 4A). Finally, the activity of ErbB2 was decreased by androgen stimulation (Fig. 2D), which was associated with concomitant decrease in the glycosylation of the receptor (Fig. 4A). This suggests that AR has effects not only on transcription and translation but also on the processing of the RTKs.

### N-linked glycosylation is required for the correct processing and localization of IGF-1R

IGF-1R is modified via N-linked glycosylation in prostate cancer cells and this strongly associates with the signalling activity of the receptor (Figs. 2D and 4A). IGF-1R is translated in a pro-receptor form that is further processed to α and β subunits [38]. We hypothesized that modification of IGF-1R would be important for its processing and stability. In order to test the importance of N-linked glycosylation for AR-dependent induction of IGF-1R, we treated LNCaP cells with androgens either in the presence or absence of tunicamycin, and found that induction of IGF-1R was blocked by 80% by tunicamycin (Fig. 4B). This suggested that N-linked glycosylation of the receptor might be essential for its stabilization. In order to get further insights into how N-linked glycosylation regulates IGF-1R, we stimulated LNCaP cells with R1881 for 48 hours and added tunicamycin for the last 18 hours. This sequential treatment with R1881 and subsequently tunicamycin meant that the overall reduction in IGF-1R expression was only 30% (Fig. 4C) in contrast to 80% reduction achieved with simultaneous treatment (Fig. 4B). In order to visualize different forms of IGF-1R in prostate cancer cells, we immunoprecipitated IGF-1R from cells stimulated with R1881 for 48 hours and probed the membrane with an antibody against IGF-1Rβ, and observed both a long form representing pro-receptor and also a band representing the IGF-1Rβ subunit (Fig. 4D). Addition of tunicamycin for the last 18 hours enhanced the mobility of pro-receptor, which suggests that processing of the receptor is altered due this treatment. The long form of the pro-receptor was decreased over 6-fold, compared to the R1881 treated condition. IGF-1R functions as a plasma-membrane receptor, which is achieved by correct processing of the pro-receptor [39].

In order to understand the importance of this altered motility, we looked at the sub-cellular localization of IGF-1R by the means of immunofluorescence. As previously reported, we detected IGF-1R at the plasma membrane and also in the cytoplasm and the nucleus [40] (Fig. 4E). Androgen stimulation led to the accumulation of IGF-1R to the plasma membrane, reflecting the increased phosphorylation of the receptor observed using the RTK array (Fig. 2D). Inhibition of N-linked glycosylation with tunicamycin impaired the accumulation of IGF-1R at the plasma membrane. By contrast we did not observe any significant changes in the staining pattern and intensity in the cytoplasm or nucleus. By taking this sequential treatment approach we were able to separate the effects on distribution and processing from the effects on expression that we observed with co-treatments.

## Discussion

In this paper we have studied the importance of glycosylation in prostate cancer cells. AR, the key transcription factor in prostate cancer regulates glycolytic metabolism and lipid, amino acid and nucleotide biosynthesis [8], [9]. Hexosamine biosynthetic pathway (HBP) requires glucose, glutamine, co-enzyme A and UTP to form UDP-GlcNAc and functions as a metabolic integration point [24], [27]. We found that two enzymes of the HBP, namely GFPT1 and UAP1 are up-regulated in prostate cancer cell lines (Fig. 1A). GFPT1 is the rate-limiting enzyme in the HBP and it has been identified as an important contributor to Kras-driven pancreatic ductal adenocarcinoma (PDAC) [41]. In PDAC, activation of Kras supports anabolic glucose metabolism and tumor growth in mice depends on the expression of GFPT1. Interestingly, AR was recently reported to activate glycolytic metabolism [8], suggesting that AR might have similar effects on metabolic control in prostate cancer to those exerted by Kras does in PDAC.

HBP provides substrates for O- and N-linked glycosylation and we found that prostate cancer cells are sensitive to inhibitors targeting both of these processes (Fig. 1B and C). This is supported by the fact that the enzyme catalyzing O-linked glycosylation is up-regulated in breast and prostate cancers and inhibition of its expression with shRNA decreases tumor growth in animal models of these cancers [31], [42].

UDP-GlcNAc can also be utilized by ER and Golgi glycosyl transferases to modify proteins destined to the plasma-membrane and secretion [24]. Increased N-linked glycosylation enhances the binding of galectins to RTKs, which in turn enhances cell-surface expression [25], [43]. In essence, HBP acts as a metabolic integration point to regulate growth promoting pathways according to the availability of energy. RTKs can support activation of AR, and antibodies targeting IGF-1R have been shown to inhibit AR activity in clinical setting [44]. We found that inhibition of N-linked glycosylation with tunicamycin inhibited the expression KLK3, a direct target of AR and biomarker used for blood-based test of prostate cancer [32]. KLK3 is glycosylated in the ER and we used other AR target proteins to see if tunicamycin affected these as well. Interestingly, we found that tunicamycin completely blocked androgen induced up-regulation of CaMKK2 and IGF-1R (Figs. 2A, 2B and 4B).

We next wanted to identify RTKs that are activated by androgen stimulation in prostate cancer cells. An antibody array recognizing phosphorylated RTKs showed that IGF-1R is activated by 10-fold upon androgen stimulation, ErbB2 activity is decreased by 3-fold while EGFR activity remains the same (Figs. 2C and 2D). Interestingly, androgen treatment induced a prominent switch in the RTK activity. Chen *et al*. used a prostate cancer mouse model to show that androgen deprivation sensitizes prostate cancer cells to the dual inhibition of EGFR and ErbB2 [45]. This is further supported by our data, since in the absence of androgens LNCaP cells largely rely on signalling via EGFR and ErbB2 (Fig. 2D). IGF-1R isup-regulated in prostate cancer [46], [47] and is knownto be up-regulated atthe mRNA and protein levels by androgen stimulation [13]. Here we show that IGF-1R is the RTK that is most activated by androgen stimulation (Fig. 2D).

The dwell-time of a given RTK on the plasma-membrane can be regulated by N-linked glycosylation, and increased glycosylation triggers galectin binding to RTKs, which stabilizes them [25], [43]. Based on lectin enrichment we found that IGF-1R glycosylation was increased by hormone stimulation 4-fold, whilst glycosylation of EGFR was only modestly increased (Fig. 4A). We used tunicamycin to inhibit N-linked glycosylation and assess the effects on IGF-1R distribution. Simultaneous treatment with androgen and tunicamycin blocked IGF-1R induction (Fig. 4B). We therefore added tunicamycin only in the end of the hormone stimulation, which ensured that IGF-1R protein was induced (Fig. 4C). In order to assess the effects on processing, we immunoprecipitated IGF-1R and found that tunicamycin treatment altered the motility of IGF-1R pro-receptor (Fig. 4D). A glycosylation site has been mapped on IGF-1R, asparagines at position 913, which when mutated blocks the trafficking of the receptor to the plasma membrane [48]. This led us to hypothesise that the tunicamycin treatment, which resulted in a modest shift in the mobility of IGF-1R in the gel through a reduction in glycosylation, might also disrupt membrane localisation of the receptor. We found that tunicamycin did indeed block androgen-induced translocation of IGF-1R to the plasma-membrane (Fig. 4E). An additional example of a requirement for N-linked glycosylation in the membrane targeting of RTKs comes from a recent study by Chen *et al*.,(2012). In this study they used tunicamycin to inhibit N-linked glycosylation and found that this to blocked the plasma-membrane localization of c-Met in hepatocellular carcinoma cells [49].

In conclusion we have established that N-linked glycosylation of IGF-1R is necessary for the full activation of the receptor in response to androgen treatment and that perturbing this process can break the feedback loop between AR and IGF-1R activation in prostate cells. Achieving similar results selectively in a clinical setting will be an important challenge for future studies.

## Supporting Information

Figure S1 **Inhibition of N-linked glycosylation affects transcription of KLK3 and CaMKK2.** LNCaP cells were deprived of androgens for 72 hours and stimulated with 10 nM R1881 either in the presence or absence of tunicamycin (5 µg/ml) for 24 hours and total mRNA was collected. KLK3, CaMKK2 and TBP (TATA-binding protein) were detected with TaqMan assays. Values were first normalized to TBP and then to the vehicle treated sample. The data shown represents values obtained from biological replicate and standard error of mean is shown.
(EPS)

Figure S2 **Androgen receptor and RNA pol II are recruited to the genomic regions of EGFR and IGF-1R in LNCaP cells.** The data for AR was obtained from Yu *et al*. (2010) and for RNA polII from Massie *et al*. (2011) [8] and is depicted as screenshots from the UCSC genome browser having been uploaded and viewed in the hg18 build for (A) EGFR and (B) IGF-1R.
(EPS)

Figure S3 **Androgen receptor is recruited to the genomic regions of EGFR and IGF-1R in VCaP cells.** The data for AR was obtained from Yu *et al*. (2010) [7] and is depicted as screenshots from the UCSC genome browser having been uploaded and viewed in the hg18 build for (A) EGFR and (B) IGF-1R.
(EPS)
